# Treatment of Exposed Pulps

**Published:** 1873-11

**Authors:** J. S. King


### TREATMENT OF EXPOSED PULPS.

BY DR. J. S. KING.

In the following case I give a treatment of exposed pulps, that has been in my hands very successful :

About two years ago I made what I deemed a discovery in the treatment of exposed and wounded tooth pulps. My success in this particular since that time, has been exceedingly gratifying to myself, and also beneficial to my patrons. I will here refer to Dr. J. Gillespie, Dr. J. C. Templeton and others of my professional friends in Pittsburg, if desired, whose experience in my mode of treatment has been as gratifying to them, as mine has been to me. I will in this article give my mode of treatment of all exposed and painful pulps, in which inflammation has not terminated in sloughing, and final death of the parts, and if you deem this article worthy of attention, you can use it as you may think best.

Perhaps I can best describe my method by citing a case or two.—Case of Miss S. J. P. Left superior central incisor, for some weeks painful, mesial cavity ; pulp exposed ; in removing the decay preparatory to treatment and filling, considerable bleeding from the pulp ensued ; after applying rubber dam, I thoroughly washed the cavity wlth a pellet of cotton, well saturated with creosote, thus cleansing all particles from the cavity, and also checking the flow of blood from the exposed and wounded pulp. I then placed on a plate of glass two parcels of Smith's white oxide of zinc ; the smaller one of these parcels I mix with creosote to the consistency of paste ; this mixture I very carefully apply in proper quantity to the exposed nerve-pulp and surrounding sensitive dentine, well covering and protecting the same (yet covering very lightly), so that my next or second covering of oxy-chloride will not at any point come in contact with the exposed pulp or surrounding sensitive tooth bone, thus avoiding all pain resulting from the direct application of oxy-chloride to exposed pulps and sensitive dentine. The first application of white oxide and creosote being now in place, by carefully compressing it with a small pellet of cotton wrapped around a small bur-head-excavator, and then removing any excess that may remain around the border of the cavity, I then mix the second parcel on the glass with the usual chloride of zinc, a little softer than the first so that it will not displace the same. With this I proceed to cover up the first application, and also to fill up the cavity to the extent I wish, and allow it to harden; after which I proceed to cut away sufficient of the oxy-chloride to give me proper retaining points for the insertion of a good gold filling. When a tooth has been painful, or the pulp largely exposed, I usually postpone the insertion of the gold filling, until after the expiration of ten days or three weeks, thus giving nature ample time for proper recuperation. This case of Miss S. J. P. was of more than usual interest to me. Some thirteen months after the insertion of said filling, I removed it and the covering, and found the pulp in good condition; it again bled freely from being wounded by my excavator. I was disappointed in not finding secondary dentine deposited as a protection to the pulp. I again immediately resorted to the former treatment, and filled once more with the same happy result.

Again in another case, I excavated a large labial cavity in a lower right third molar. Pulp exposed and bled slightly; treated as described in the foregoing of this article; patient called several days afterwards, tooth entirely comfortable. I expect to finish it with gold in a few days.

In another case, a gentleman called and asked the extraction of a lower left second molar. I examined the case, and found a large distal cavity and exposed pulp. I declined to extract; I cleansed the cavity, had no bleeding of the pulp. I treated according to my usual custom; I have no doubt of the result.

